# American Dental Association

**Published:** 1868-09

**Authors:** 


### AMERICAN DENTAL ASSOCIATION.

THE eighth annual meeting of the American Dental Association was held at Niagara Falls, beginning the 28th of July, and continuing four days. It was, probably, the largest meeting of that body ever held: there was about two hundred members in attendance.

The meeting in many respects was a profitable one. The first two days was occupied in business of a miscellaneous character. Matters were presented upon which there was considerable diversity of opinion, and about which it seemed necessary that free expression should be given, in order that correct conclusions might be attained, and we are pleased to know that something—yes much—was accomplished. In those discussions were elaborated the principles that in future will govern the Association. Each year new points arise and are settled. He who cannot see that progress is being made is blind indeed. Much discussion was had upon the character of the representation that should be admitted, a point upon which the efficiency, and even the life of the institution depends ; for looseness and vagueness in this respect would always be productive of evil, and the time had arrived when that must be definitely settled. We have little sympathy with those who become disgusted with the settlement of so important matters.

The following amendment to the constitution, which was proposed two years ago, and last year was vigorously opposed, and laid upon the table, was now taken up and passed without dissent, viz: "To be entitled to representation in this Association, Dental Societies shall be required to adopt or substantially recognize its code of Ethics." Also, the amendment proposed two years ago, viz: "Any member of a local or state association, who is expelled from the same, shall cease to be a member of this Association from the date of his expulsion," was taken up and adopted without dissent. The passage of these amendments certainly indicates progress onward and upward.

It has been a matter of regret heretofore that there seemed to be so little disposition to decide upon the policy and principles by which the Association should be governed. Lax and indefinite rules and regulations, are always productive of confusion; an illustration of which we have had in the American Dental Association for the past three or four years.

As a result of the discussions of the second day, a committee was appointed to revise the Constitution, By-laws and Order of Business, and so thoroughly imbued were they with the necessity of something being done, that they immediately proceeded to the discharge of this duty, and were able to present a complete report before the final adjournment. In reference to the Constitution and By-laws action could not be had till the next annual meeting, but the order of business presented was adopted, and will be the order for the next annual meeting. The principal change made in this is putting the principal part of the miscellaneous business, including the election of officers, near the close, instead of the beginning of the session, so that immediately after the necessary business of the organization, embracing the report of the committee on membership, the reports of standing committees, and discussions upon the same will be in order.

The reports of standing committees should be read, and the length of time appropriated to their consideration should be designated, and a proper division of time given to each subject. Heretofore, the consideration of the first subjects presented has consumed by far too much time; or, more than their proper proportion of time.

The Association adjourned to meet at Saratoga Springs on the first Tuesday of August, 1869, at 10 A. M. T.

